# Socioemotional Resources Account for Academic Adjustment in Moroccan Adolescents

**DOI:** 10.3389/fpsyg.2020.01609

**Published:** 2020-07-03

**Authors:** Daniel Cortés-Denia, Karima El Ghoudani, Manuel Pulido-Martos, Smail Alaoui, Octavio Luque-Reca, Manuel Miguel Ramos-Álvarez, José María Augusto-Landa, Benaissa Zarhbouch, Esther Lopez-Zafra

**Affiliations:** ^1^Departamento de Psicología, Psicología Social, Universidad de Jaén, Jaén, Spain; ^2^Ecole Supérieure d’Education et de Formation (ESEF), Psychologie, Hassan First University of Settat, Settat, Morocco; ^3^Département de Psychologie, Faculté des Lettres et des Sciences Humaines – Dhar El Mahraz, Université Sidi Mohamed Ben Abdellah, Fez, Morocco; ^4^Departamento de Psicología, Universidad Rey Juan Carlos, Madrid, Spain; ^5^Departamento de Psicología, Metodología de las Ciencias del Comportamiento, Universidad de Jaén, Jaén, Spain

**Keywords:** academic performance, adolescents, anxiety, depression, emotional intelligence, self-concept, Morocco, social support

## Abstract

This study aimed to analyze the relationship of both positive socioemotional resources [emotional intelligence (EI) and social support] and negative states (test anxiety and depression) with academic adjustment, as measured by academic performance and self-concept, among Moroccan adolescents. The participants were 845 students from Morocco (372 boys, 473 girls; mean age 15.58 years; SD = 1.69; range = 13–18) who were attending secondary education (79.8%) or high school. The participants completed a questionnaire that included scales to measure the variables of interest, adapted for and validated in Moroccan adolescents. A multiple mediation serial model with four mediator variables confirmed that academic self-concept was positively and directly predicted by EI, academic performance, and social support, whereas test anxiety and depression had a negative effect. Second, EI predicted self-concept through its indirect effects on test anxiety and academic performance, social support, and depression. EI was the most protective factor. This model has good performance in explaining the variation in test anxiety (1.6%), depression (14.2%), social support (9.5%), academic performance (6.8%), and self-concept (35.7%). This study helps clarify the relationship of positive and negative socioemotional states with the academic performance of adolescents in Morocco. This study contributes to the literature by enhancing knowledge of adolescents in societies that, like Morocco, have a less elaborated tradition at these levels of education and that are considering education in their agenda as a way of enhancing national development and promoting EI to allow youth development in a healthier society.

## Introduction

For Arab countries, including Morocco, studies on socioemotional and academic adjustment, especially studies focusing on adolescents, are still scarce. The linguistic conception of adolescence as not an age but rather a transition stage based on biology and the lack of instruments validated in the Moroccan context to measure these aspects (Zarhbouch, 2020) have provoked a gap in our understanding of this life stage. Moreover, assessments of the relation between adolescence and educational context in Morocco face an added difficulty due to the strong family and social constraints faced by adolescents, especially girls (Tazi, 2008). Previous studies that have considered Moroccan adolescents have focused on immigrants living in other, mainly European countries (Curtis et al., 2018), but recently, Morocco has displayed an interest in using the education system to enhance its development (World Bank Group, 2019), prompting growth in efforts to analyze which areas should be focused on. These areas include physical and mental health, emphasizing medical models (i.e., Zouini et al., 2019a, b), and psychosocial aspects [i.e., social support or emotional intelligence (EI); Lopez-Zafra et al., 2019]. However, the interplay between these psychosocial (socioemotional) aspects and academic adjustment still needs to be addressed. Thus, in this study, we propose to deepen the understanding of these relations by analyzing the impact that protective (social support and EI) and negative (depression and test anxiety) factors have on academic adjustment (academic self-concept and academic performance), thereby proposing a comprehensive model of relations to further our knowledge of Moroccan adolescents.

Our starting point is the study by Lopez-Zafra et al. (2019), in which the authors prove that social support and EI are protective factors for well-being, positively increasing life satisfaction and reducing depression. Here, we further test whether socioemotional adjustment relates to academic adjustment in Moroccan adolescents.

Previous studies in other countries have shown that socioemotional variables are more relevant than cognitive skills for academic success (see the review by Ambiel et al., 2015). Indeed, socioemotional variables are recognized as important in the academic context and are associated not only with academic success (MacCann et al., 2020) but also with success in life (Lussier and Fitzpatrick, 2016). Among these variables, EI has been found to be related to adolescents’ psychological adjustment (Resurrección et al., 2014) and academic performance (MacCann et al., 2020). However, the latter relation is usually indirect, involving the promotion of other positive results (i.e., social attitudes; Jiménez and Lopez-Zafra, 2011), influencing academic performance due to a better cope with stress (Extremera and Fernández-Berrocal, 2004). Thus, EI may mediate the relation between mental health and academic performance and further analyses of the possible variables involved are needed.

Regarding academic adjustment, the notion of academic self-concept involves one’s emotional and cognitive self-evaluation (see Hattie, 2014) and relates to success and academic performance at all levels of education (Marsh et al., 2016). Indeed, studies have confirmed the positive relation between academic self-concept and academic performance (see Barker et al., 2005). However, there are doubts about the direction of causality. There are studies indicating that self-concept is reinforced by academic performance (Peralta Sánchez and Sánchez Roda, 2003), whereas other studies consider the relation to be the other way around (Ferla et al., 2009). Thus, in this study, we also analyze the direction of this relation.

Academic performance may be reduced due to negative factors. For example, adolescents have to cope with test anxiety and depression, which are relatively common at this stage (Foster et al., 2018). Test anxiety is defined as a set of physiological, behavioral, and cognitive responses related to an individual’s feelings over his/her expected exam performance (Nicaise, 1995), and it is associated with lower academic performance in all cultures (Raymo et al., 2019). However, personal and socioemotional variables also interact in this relation, which may be attenuated by EI or social support. In fact, EI is negatively related to depression and anxiety (Fernández-Berrocal et al., 2006) and has been proven to act as a protective factor jointly with social support in determining levels of life satisfaction in Moroccan adolescents (Lopez-Zafra et al., 2019).

Bearing all these aspects in mind, we propose and test the following hypotheses.

Hypothesis 1. EI is positively related to academic self-concept.
Hypothesis 2. Academic performance positively predicts academic self-concept.
Hypothesis 3. The relation of EI with anxiety affects academic performance and self-concept, leading to an improvement in academic performance and, therefore, academic self-concept.
Hypothesis 4. The positive impact of EI on social support produces lower levels of depression and an improved academic self-concept.

In sum, our study focuses on the role that socioemotional variables such as social support, EI, test anxiety, and depression play in academic adjustment, measured by academic performance and self-concept, among Moroccan adolescents. This is a novel approach to a sample that has received little attention.

## Materials and Methods

### Participants and Procedure

The participants in this study were 845 students from Morocco (372 boys, 473 girls; mean age 15.58 years; SD = 1.69; range = 13–18) who were attending secondary education (*n* = 675) or high school (*n* = 168), with two missing values. The participants completed a questionnaire with scales measuring the variables of interest, adapted for and validated in Moroccan adolescents.

The Research and Ethics Committee at the Faculty of Letters and Human Sciences-Dhar el Mehraz of the University of Sidi Mohamed Ben Abdellah in Fez (Morocco) and the Regional Academy of Education and Training gave ethical support and allowed access to public schools. Administrative and education officials approved the questionnaire and procedure to be administered in public schools and gave the researchers a letter to present in the schools. All schools in the region of Fez – Mequinez were invited to participate. In those accepting, students’ families were informed about the study with a written letter explaining the research to obtain verbal parental consent for all participants. If parents did not consent, they could return the letter with a request to be excluded. All parents agreed to allow their children to participate, and the schools reported this information to the researchers. The Ethics Committee approved all consent procedures.

The questionnaires were administered by a group of 12 assistants who had received previous instructions about the scales, meanings of items, and procedure. They were also instructed to follow the ethical procedure guidelines approved by the Ethics Committee and the Regional Academy of Education and Training. Inclusion criteria were to be Moroccan, student in public school, and consent to participate. Participants over 18 years old were excluded.

### Measures^1^

#### Wong and Law Emotional Intelligence Scale

The WLEIS (WLEIS; Wong and Law, 2002; moroccan version by El Ghoudani et al., 2020a; α = 0.79 and adequate validity), is a short instrument comprising 16 items scored on a 7-point Likert scale. The Moroccan version comprises 15 items measuring four competencies: self-emotional appraisal, emotional appraisal of others, use of emotions, and regulation of emotions. A higher score indicates that an adolescent is more able to perceive/assess, use and regulate his/her emotions and those of others.

#### Multidimensional Scale of Perceived Social Support

We use the Multidimensional Scale of Perceived Social Support Arabic Language – Moroccan Adolescents (MSPSS. AL-MA). This is the adaptation of the Multidimensional Scale of Perceived Social Support – Arab Adolescents by Ramaswamy et al. (2009) for Moroccan adolescent samples (El Ghoudani et al., 2020b; α = 0.65, similar to the original, and adequate validity). This version is composed of 12 items with a dichotomous response format (Yes or No). The scale reliability indices are similar to those of Ramaswamy et al. (2009), which is based on the original scale by Zimet et al. (1988; see Cortés-Denia et al., 2020). The instrument assesses the social support that an adolescent receives from family, friends and school personnel.

#### Test Anxiety Scale

The Test anxiety scale, Arabic Language – Moroccan Adolescents (Sarason TAS. AL-MA by Zarhbouch et al., 2020a, α = 0.96 and adequate validity), is an adaptation of the original Test Anxiety Scale for Children (TASC; Sarason et al., 1960). This Moroccan version is composed of 38 items answered on a 4-point Likert scale (from 4, totally disagree, to 1, totally agree) that measures one factor, namely, anxiety caused by academic evaluations of performance.

#### Academic Performance

To obtain objective and comprehensive data to evaluate academic performance, students’ grade was considered. This grade is the weighted average of the grades obtained in all subjects, where the degree of relevance of each subject changes depending on the specialty. The range is 20 points, with a grade under 10 indicating failure of the academic year and a grade over 17 indicating excellence. In our sample, the range was from 5 to 19.99 points, with a mean grade of 12.88 (SD = 2.67).

#### Self-Concept of Academic Ability Scale

This Arabic Language – Moroccan Adolescents scale (SCAAS. AL-MA by Zarhbouch et al., 2020b; α = 0.85 and adequate validity), is based on Aal-Hussain (1991), who adapted that of Brookover et al. (1962) into Arabic. It is composed of 8 items scored on a Likert scale (from 1, very poor, to 4, outstanding), distributed over two factors: general, that is, the individual’s ability to complete their studies in absolute terms and in comparison with the ability of other students (5 items); and undergraduate, that is, the expectations an individual has in relation to the university context (3 items). In this study, participants are from secondary and high school, and thus, we focus only on the general self-concept.

#### Beck Depression Inventory-II

The BDI-II (Beck et al., 1996) measures the severity of depression through a series of symptoms related to different depressive disorders. The Moroccan version is a 19-item inventory that assesses depressive symptomatology (Alaoui et al., 2020; α = 0.88 and adequate validity). Each item is rated on a scale ranging from 0 (normal) to 3 (most severe), with summary scores ranging between 0 and 57. The BDI-II has yielded high internal consistency among college students (α = 0.93; Beck et al., 1996).

### Data Analysis

IBM SPSS v. 22 was used to compute descriptives (means, standard deviations, reliability, asymmetry, and kurtosis) and Pearson correlations. PROCESS v. 3.4 (Hayes, 2018) with a confidence interval (CI) of 95% was used to perform multiple mediation in series and in parallel.

## Results

### Descriptive Statistics

Table 1 shows the means, standard deviations, internal consistency, and correlations among all the variables of the study. All scores were normally distributed as indicated by asymmetry and kurtosis. The Pearson correlation coefficients showed that EI related positively to social support, self-concept, and academic performance, whereas the relation with anxiety and depression was negative. Similarly, self-concept displayed a positive relation with social support and academic performance and a negative relation with test anxiety and depression.

**TABLE 1:** Descriptive statistics and correlations among the main variables.

	Mean	SD	α	Skewness	Kurtosis	1	2	3	4	5	6
1. Emotional intelligence	3.11	0.44	0.80	–0.67	1	–					
2. Test anxiety	2.70	0.62	0.96	–0.34	–0.20	−0.12***	–				
3. Academic performance	12.88	2.67	–	–0.01	–0.36	0.10**	−0.25***	–			
4. Social support	1.61	0.19	0.62	–0.30	0.33	0.32***	–0.03	0.08*	–		
5. Depression	2.15	1.37	0.89	0.93	–0.29	−0.33***	0.32***	−0.18***	−0.28***	–	
6. Academic self-concept	3.04	0.49	0.85	–0.25	0.82	0.31***	−0.28***	0.50***	0.22***	−0.29***	–

*Note.*α indicates the Cronbach’s alpha value for the reliability of the questionnaire; * *p* < 0.05; ** *p* < 0.01; *** *p* < 0.001.

### Multiple Mediation Analyses in Series and in Parallel

To examine the implications of EI for academic self-concept and the existence of possible mediating variables that could influence this relationship, a multiple and serial mediation model of four moderating variables was tested with the PROCESS macro (Model 82; Hayes, 2018), where EI acted as a predictor and academic self-concept as a result variable, with anxiety, academic performance, social support, and depression analyzed as possible mediating variables. For the analysis, 10,000 bootstraps, Davidson–Mackinnon’s heteroscedasticity-consistent inference, and a CI of 95% were used. The results showed a significant total effect of EI on self-concept (β = 0.31, SE = 0.04, *p* < 0.001, CI 95% [0.270, 0.433]). Furthermore, the direct effect of EI on self-concept remains significant, even considering anxiety, academic performance, social support and depression (β = 0.19, SE = 0.04, *p* < 0.001, CI 95% [0.138, 0.289]) (see Table 2).

**TABLE 2:** Parallel and serial multiple mediation model: Total, direct, and indirect effects between emotional intelligence and self-concept through anxiety, academic performance, social support, and depression.

	β	SE	*p*	95% CI [lower, upper]
Total	0.31	0.04	<0.001	[0.270, 0.433]
Direct	0.19	0.04	<0.001	[0.138, 0.289]
	**Std. Effect**	**Bootstrap SE**	***P***	**Bootstrap 95% CI [lower, upper]**
Indirect total	0.12	0.02	–	[0.076, 0.165]
Ind1: X→M1→Y	0.02	0.01	–	[0.004, 0.028]
Ind2: X→M2→Y	0.03	0.02	–	[0.001, 0.060]
Ind3: X→M3→Y	0.03	0.01	–	[0.008, 0.056]
Ind4: X→M4→Y	0.03	0.01	–	[0.008, 0.044]
Ind5: X→M1→M2→Y	0.01	<0.01	–	[0.006, 0.023]
Ind6: X→M3→M4→Y	0.01	<0.01	–	[0.002, 0.011]

**Note.* Model 82 (four mediators). β = standardized coefficients; bootstrap = 10,000; CI = confidence interval; M1 = anxiety; M2 = academic performance; M3 = social support; M4 = depression; SE = standard error; X = emotional intelligence; Y = self-concept.*

**FIGURE 1:**
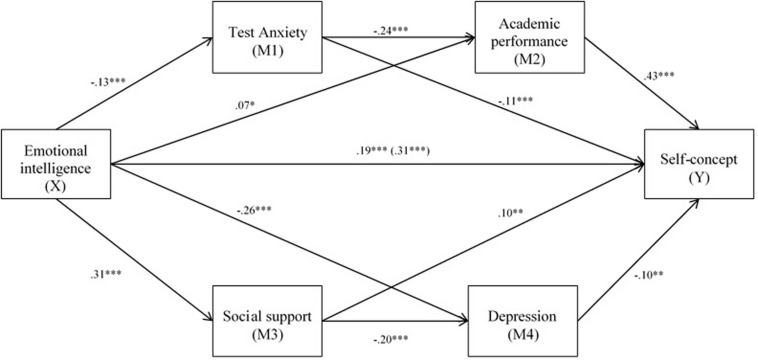
Results of model of multiple mediation in series and in parallel. *Note.* Values are standardized coefficient. The values in parentheses represent the total effect. ^∗^*p* < 0.05; ^∗∗^*p* < 0.01; ^∗∗∗^*p* < 0.001.

Moreover, EI negatively and significantly influenced anxiety (β = −0.13, SE = 0.05, *p* < 0.001) and depression (β = −0.26, SE = 0.12, *p* < 0.001) and positively influenced academic performance (β = 0.07, SE = 0.21, *p* < 0.05) and social support (β = 0.31, SE = 0.02, *p* < 0.001). In a similar vein, self-concept was negative and significantly predicted by anxiety (β = −0.11, SE = 0.03, *p* < 0.001) and depression (β = −0.10, SE = 0.01, *p* < 0.01) and positively predicted by academic performance (β = 0.43, SE = 0.01, *p* < 0.001) and social support (β = 0.10, SE = 0.09, *p* < 0.01). In turn, significant and negative implications of anxiety were found for academic performance (β = −0.24, SE = 0.16, *p* < 0.001) and of social support for depression (β = −0.20, SE = 0.26, *p* < 0.001) (see Figure 1 and Table 3).

**TABLE 3:** Relationships between model variables.

	Consequent														
	M1			M2			M3			M4			Y		
Antecedent	β	SE	*P*	β	SE	*p*	β	SE	*p*	β	SE	*p*	β	SE	*p*
X	−0.13	0.05	<0.001	0.07	0.21	<0.05	0.31	0.02	<0.001	−0.26	0.12	<0.001	0.19	0.04	<0.001
M1	–	–	–	−0.24	0.16	<0.001	–	–	–	–	–	–	-0.11	0.03	<0.001
M2	–	–	–	–	–	–	–	–	–	–	–	–	0.43	0.01	<0.001
M3	–	–	–	–	–	–	–	–	–	-0.20	0.26	<0.001	0.10	0.09	<0.01
M4	–	–	–	–	–	–	–	–	–	–	–	–	-0.10	0.01	<0.01
Constant	*3.27	0.17	<0.001	*14.36	0.86	<0.001	*1.18	0.06	<0.001	*6.99	0.48	<0.001	*1.25	0.20	<0.001
	*R*^2^ 0.016 *F*(1,820) = 11.448, *p* < 0.001			*R*^2^ 0.068 *F*(2,819) = 27.392 *p* < 0.001			*R*^2^ 0.095 *F*(1,820) = 58.870 *p* < 0.001			*R*^2^ 0.142 *F*(2,819) = 55.448 *p* < 0.001			*R*^2^ 0.357 *F*(5,816) = 76.471 *p* < 0.001		

*Note.* β = standardized coefficient; M1 = anxiety; M2 = academic performance; M3 = social support; M4 = depression; SE = standard error; X = emotional intelligence; Y = self-concept; * unstandardized coefficient.

This model shows the significant relationships among the six indirect effects connecting EI and self-concept, specifically anxiety (M1; 95% CI [0.004, 0.028]), academic performance (M2; 95% CI [0.001, 0.060]), social support (M3; 95% CI [0.008, 0.056), depression (M4; 95% CI [0.008, 0.044]), and serial paths from M1 to M2 (95% CI [0.006, 0.023]) and M3 to M4 (95% CI [0.002, 0.011]) when zero is not included within the CI (see Table 2). This model explains 1.6% of the variation for test anxiety, 14.2% for depression, 6.8% for academic performance, 9.5% for social support, and 35.7% for self-concept.

## Discussion

Due to the importance of understanding socioemotional and academic adjustment for the future of Moroccan adolescents, our motivation for testing a model of relations is clear. This study contributes by considering both protective (EI and social support) and negative (anxiety and depression) variables affecting academic performance in the same model. Furthermore, our results contribute to the Moroccan educational system by suggesting the inclusion of emotional abilities in the educational process.

The positive relationship between EI and academic self-concept (H1) is a novel result, as most studies have focused on the impact of EI on academic performance (MacCann et al., 2020). Moreover, academic performance mediates this relation, confirming that EI is a predictor of academic success and supporting the results of other studies (Romanelli et al., 2006). However, the path of relations in this study implies that self-concept results from academic performance to a greater extent than the other way around, also confirming that our H2 that is in line with social identity theory, in which positive comparisons in results improve adolescents’ self-concept (Sinclair et al., 2019).

Furthermore, anxiety and depression were included, as they might mediate this relation due to their proven negative relation with self-concept (Najafi Kalyani et al., 2013). Indeed, our results showed that greater EI reduces anxiety. Moreover, anxiety mediates the relation between EI and self-concept, therefore supporting H3, in which the impact of EI on anxiety promotes an improvement in academic performance and, in turn, self-concept. This result is consistent with all studies that highlight the protective effect of EI in stressful situations (Resurrección et al., 2014). This result supports the importance of intervention programs for adolescents with clear implications for educational practice. Finally, other studies finding that EI and social support prevent depression (Lopez-Zafra et al., 2019) are extended by our finding that this effect also influences self-concept by reducing the negative impact of depression, thus supporting H4. This result in Moroccan students is in line with results in other countries reported by MacCann et al. (2020) in which indicate that the contribution of EI in the regulation of negative emotions (i.e., anxiety or boredom) is responsible of the positive effects of EI on academic performance.

In sum, our hypotheses are mainly supported, showing a model of relations that include positive and negative variables impacting self-concept (see Figure 1). This relational model can help researchers and practitioners address academic adjustment by considering the importance of socioemotional adjustment among Moroccan adolescents. Promoting interventions that enhance EI, as a clear predictor of self-concept, and social support from families and schools, as protective resources against anxiety and depression, will have benefits for academic performance and self-concept. Overall, the education system should attend to socioemotional aspects to enhance academic adjustment (performance and self-concept) among Moroccan adolescents.

In terms of limitations, the use of a convenience sample and the transversal design prevents us from widely generalizing the results and points to the need for longitudinal studies to further analyze the mechanisms of the EI/performance relationship. For example, MacCann et al. (2020) suggest analyzing this further with research on (a) social relationship building, (b) regulation of academic emotions, and (c) content overlap between EI and academic subject matter. However, regarding the sampling method, our sample is large and heterogeneous in terms of academic performance and the scores on the different scales, which is a strength in assessing the impact of psychological and socioemotional variables.

## Data Availability Statement

The raw data supporting the conclusions of this article will be made available by the authors, without undue reservation.

## Ethics Statement

The studies involving human participants were reviewed and approved by the Research and Ethics Committee at the Faculty of Letters and Human Sciences-Dhar el Mehraz of the University of Sidi Mohamed Ben Abdellah in Fez (Morocco). Written informed consent from the participants’ legal guardian/next of kin was not required to participate in this study in accordance with the national legislation and the institutional requirements.

## Author Contributions

All authors provided substantial contributions to the work. EL-Z, MP-M, KE, JA-L, and BZ conceived of and designed the study. BZ, KE, and SA adapted the scales to the Moroccan context, trained the surveyors, and collected the data. MR-Á performed the measurements. DC-D processed the data, performed the analyses, interpreted the data, and helped with the references. MP-M, JA-L, and OL-R contributed to the conceptual model. DC-D, EL-Z, and KE drafted the manuscript. EL-Z integrated and coordinated the work. All authors critically revised the manuscript, approved this version, and agreed to be accountable for all aspects of this work and its integrity.

## Conflict of Interest

The authors declare that the research was conducted in the absence of any commercial or financial relationships that could be construed as a potential conflict of interest.

## Footnotes

1 All measures are fully described in *Escalas de evaluación psicológica: adaptación cultural y validación psicométrica al ámbito educativo. En lengua árabe para adolescentes marroquíes*, eds B. Zarhbouch and K. El Ghoudani (Fez: Université Sidi Mohamed Ben Abdellah). All yielded adequate psychometric properties and validation.
